# Snake venom induces an autophagic cell death via activation of the JNK pathway in colorectal cancer cells

**DOI:** 10.7150/jca.75791

**Published:** 2022-09-21

**Authors:** Ji Eun Yu, In Jun Yeo, Dong Won Lee, Ju Young Chang, Dong Ju Son, Jaesuk Yun, Sang-Bae Han, Jin Tae Hong

**Affiliations:** College of Pharmacy and Medical Research Center, Chungbuk National University, 194-31, Osongsaengmyeong 1-ro, Osong-eup, Cheongju-si, Chungbuk 28160, Republic of Korea.

**Keywords:** Snake venom, Autophagy, LC3, JNK

## Abstract

Snake venom contains many proteins that help treat or prevent thrombosis, cardiovascular disease, and cancer, and many studies have been reported in this regard. It has recently been reported that autophagy exerts anticancer effects by inducing tumor cell death and inhibiting cell growth. In this study, we investigated the effect of snake venom on autophagy. Unlike normal colon cells, LC3-II protein levels and LC3 puncta accumulation are increased in snake venom-treated colorectal cancer cells. Inhibition of autophagy by treating cells with hydroxychloroquine, an autophagy inhibitor, prevented snake venom-induced cell death, indicating that snake venom indeed induces autophagic cell death in human colorectal cancer cells. In addition, we demonstrated that activated JNK, and not mTOR signaling, is an upstream effector controlling autophagy. Pretreatment with SP600125, a JNK inhibitor, reversed snake venom-induced autophagy and cell death, indicating that JNK plays a critical role in snake venom-induced autophagy. This study demonstrated that snake venom can function as an anticarcinogenby induction autophagy.

## Introduction

Snake venom is highly toxic, and it was thus difficult to use it as a medicine in the past. Currently, the humankind is developing various new drugs and therapeutic agents using snake venom. With the development of acupuncture technology, which has reduced toxicity and better therapeutic effects, snake venom is now recognized as a treatment for diseases associated with severe pain, including rheumatism, shingles, atopic dermatitis, and arthritis 1-4. Compared with other treatments, treatment using snake venom is more effective and rapid, with excellent immunotherapeutic and pain-relieving effects. Moreover, snake venom possesses anti-carcinogenic effects 5-8. In our pervious study, we reported that snake venom can inhibit the proliferation of colorectal cancer cells (CRCs) and promote apoptosis, thereby confirming its anticarcinogenic activity.

Colorectal cancer accounts for approximately 9.4% of all cancer cases worldwide and is the fourth and third most common cancer in men and women, respectively. Approximately 1 million colorectal cancer cases are reported every year, and around 500,000 people die of colorectal cancer annually. In recent years, the number of patients with colorectal cancer has gradually increased. The incidence of cancer is associated with imbalances in cell production and cell death via apoptosis 9, 10. Apoptosis refers to programmed cell death; this process contributes to the normal development of each body organ by eliminating cancer cells from organisms. Cell death is largely classified into apoptosis, necrosis, and autophagy. Autophagy and apoptosis occur in cells through various stress pathways. Autophagy and apoptosis regulate the conversion of cells in intracellular protein organelles, and many related associations have been reported 11-14. Autophagy is abnormally increased or decreased in various diseases, including cancer in which it is ambivalent. The adverse effects of autophagy in cancer treatment include helping the cancer cells survive extreme conditions, for example metastatic cancer cells that lack nutrition; radiotherapy; or chemotherapy. However, autophagy continues to occur in cancer cells, which can cause cancer cell death soon after excessive degradation 13, 14. Thus, induction or inhibition of autophagy enables the treatment of several diseases, including cancer. Therapeutics, including rapamycin (a representative autophagy inducer) and its derivatives (CCl-779 and RAD001) are currently being tested in clinical trials 11, 15, 16. It is important to find a compound that has autophagic and apoptotic roles in cancer cells and analyze its specificity and mechanism; this will help identify anticancer drugs with autophagic activity that can play ambivalent roles in controlling cancer. In our previous study, we found that snake venom induces apoptosis in human CRCs 6. In the present study, we aim to investigate if snake venom can induce autophagy and assess its role in snake venom-induced cell death as well as understand the underlying molecular mechanisms.

## Materials and methods

### Cell culture and treatment

SW480 CRCs, A549 lung cancer cells, HeLa CCL2 cervical cancer cells, and U-2 OS osteosarcoma cells were obtained from the American Type Culture Collection (Manassas, VA). CCD-18Co cells were obtained from the Korean Cell Line Bank (Seoul, Korea).The SW480, CCD-18Co and HeLa CCL2 cells were cultured in Dulbecco's modified Eagle medium supplemented with 10% fetal bovine serum (FBS), 100 μg/ml penicillin, and 100 μg/ml streptomycin. A549 cells were cultured in RPMI 1640 medium supplemented with 10% FBS, 100 μg/ml penicillin, and 100 μg/ml streptomycin. U-2 OS cells were cultured in McCoy's 5A medium supplemented with 10% FBS, 100 μg/ml penicillin, and 100 μg/ml streptomycin. The cultured cells were incubated under a humidified atmosphere of 5% CO_2_ at 37 °C.

### Cell lysates preparation and western blotting

Cells were harvested with using 1x cold-phosphatate-buffered saline (PBS) and lysed with lysis buffer containing 20 mM Tris-HCl pH 7.8, 0.1% NP-40, 200 mM NaCl, 2 mM EDTA, 5 mM EGTA with protease inhibitor, and phosphatase inhibitor. The obtained cell lysates were centrifuged at 13,000 x *g* for 20 min at 4°C. In total, 10 μg of proteins was subjected to sodium dodecyl sulfate-polyacrylamide gel electrophoresis for separation and then transferred on PVDF membranes. The membranes were first incubated with specific primary antibodies and subsequently with HRP-conjugated secondary antibodies. The desired proteins were detected using an enhanced chemiluminescence substrate (#WBKLS0500, Millipore, Billerica, MA) and visualized using the FUSION Solo S chemiluminescence detection system (Vilber Lourmat, Collégien, France). The primary antibodies were as follows: anti-LC3 (#2775, CST, Beverly, MA), anti-ATG5 (#12994, CST), anti-p62 (#8025, CST), anti-beclin-1 (#3738, CST), anti-phospho-mTOR (#2971, CST), anti-mTOR(#2972, CST), anti-phospho-4E-BP1 (#2855, CST), anti-4E-BP1 (#9644, CST), anti-phospho-p70 S6 kinase (#9205, CST), anti-S6 kinase (#9202, CST), anti-phospho-ULK (#A90736, Abclonal, Wuhan, China), anti-ULK (#A8529, Abclonal), anti-cleaved caspase-3 (#9661, CST), anti-caspase-9 (#9502, CST), anti-Bcl-2 (#sc-7382, Santa Cruz, Dallas, TX), anti-Bax (#sc-7480, Santa Cruz), and anti-β-actin (sc-517582, Santa Cruz).

### Immunocytochemistry

The cells were fixed using 4% paraformaldehyde in PBS for 15 min at room temperature. The cells were then permeabilized with 100% cold-methanol for 5 min and blocked using 4% bovine serum albumin in PBS with 0.1% Triton X-100 for 1 h. The primary antibodies were added, and the cells were incubated overnight at 4°C; then, Alexa Fluor 488 (#A32723, #A32731, Invitrogen, Carlsbad, CA) or Texas Red (#T-862, #T-2767, Invitrogen)-conjugated secondary antibodies were added and the cells were incubated for 1h at room temperature. The cells were then incubated with 1 μg/ml 4',6-diamidino-2-phenylindole (#D9542, Sigma-Aldrich, St. Louis, MO) for 5 min at room temperature and subsequently mounted using the Fluoromount-G Mounting Medium (#0100-01, Southern Biotech, Birmingham, AL). The cells were visualized using the ZEISS Axio Observer fluorescence microscope system (Carl Zeiss, Oberkochen, Germany). Digital images were analyzed using ZEN 2.1 software (Carl Zeiss). For quantitative analysis, 100 cells were counted in 10 random fields and performed at least 3 independent experiments. The size of the autophagosome was 0.5 to 2 μm, and the number of LC3 puncta (0.5 to 2 μm puncta size) per cell was counted.

### Autophagy flux assay

To analyze the autophagy flux, RFP-GFP-LC3 cells were generated using the Premo™ Autophagy Tandem Sensor RFP-GFP-LC3B Kit (Thermo Scientific, Waltham, MA) according to the manufacturer's protocol. When RFP-GFP-LC3 moves to lysosomes, acid-sensitive GFP signals are quenched by pH changes, while acid-insensitive RFP signals are maintained. Using this color change, the co-expression (yellow) of GFP and RFP represents autophagosome, and the expression of RFP represents autolysosomes. Cells were transduced with tandem reagent for 24 h at 37°C. The cells were then visualized the ZEISS Axio Observer fluorescence microscope system (Carl Zeiss). Digital images were analyzed using ZEN 2.1 software (Carl Zeiss). For quantitative analysis, 100 cells were counted in 10 random fields and performed at least 3 independent experiments. The the ZEN 2.1 software was used to obtain the number of yellow puncta (RFP+GFP; autophagosome) per cell and the number of red puncta (RFP; autolysosome) per cells were obtained.

The formation of autophagosomes was assessed using the CYTO-ID^®^ Autophagy detection kit (Enzo Life Sciences Inc., Farmingdale, NY), according to the manufacturer's protocol. In brief, the cells were stained with CYTO-ID for 1 h at 37°C in the dark. The cells were then visualized the ZEISS Axio Observer fluorescence microscope system (Carl Zeiss). Digital images were analyzed using ZEN 2.1 software (Carl Zeiss).

### Cell viability assay

The cells were plated onto 96-well plates, and treated with 0, 0.1, 0.5, and 1 μg/ml snake venom for 24 h. After 24 h, cell viability was assessed using the 3-(4,5-dimethyl-2-thiazolyl)-2,5-diphenyl-2H-tetrazolium bromide (MTT) assay (#M5655, Sigma Aldrich), according to the manufacturer's protocol. In brief, 5 μg/ml MTT solution was added to the cells and the plate was incubated at 37°C for 4 h, following which 100 μL dimethyl sulfoxide was added to each well. The absorbance values of each cell sample were read using a microplate reader at a wavelength of 540 nm.

### Statistical analysis

Data were analyzed using GraphPad Prism 5 software. The error bars represent the standard deviations (SDs), unless indicated otherwise. Pairwise comparisons were performed using Student's *t*-test. Multiple comparisons were performed using one-way analysis of variance followed by Tukey's test. Differences between groups were considered significant at *P* < 0.05.

## Results

### Snake venom induces autophagy in human colorectal cancer cells

**Figure 1:**
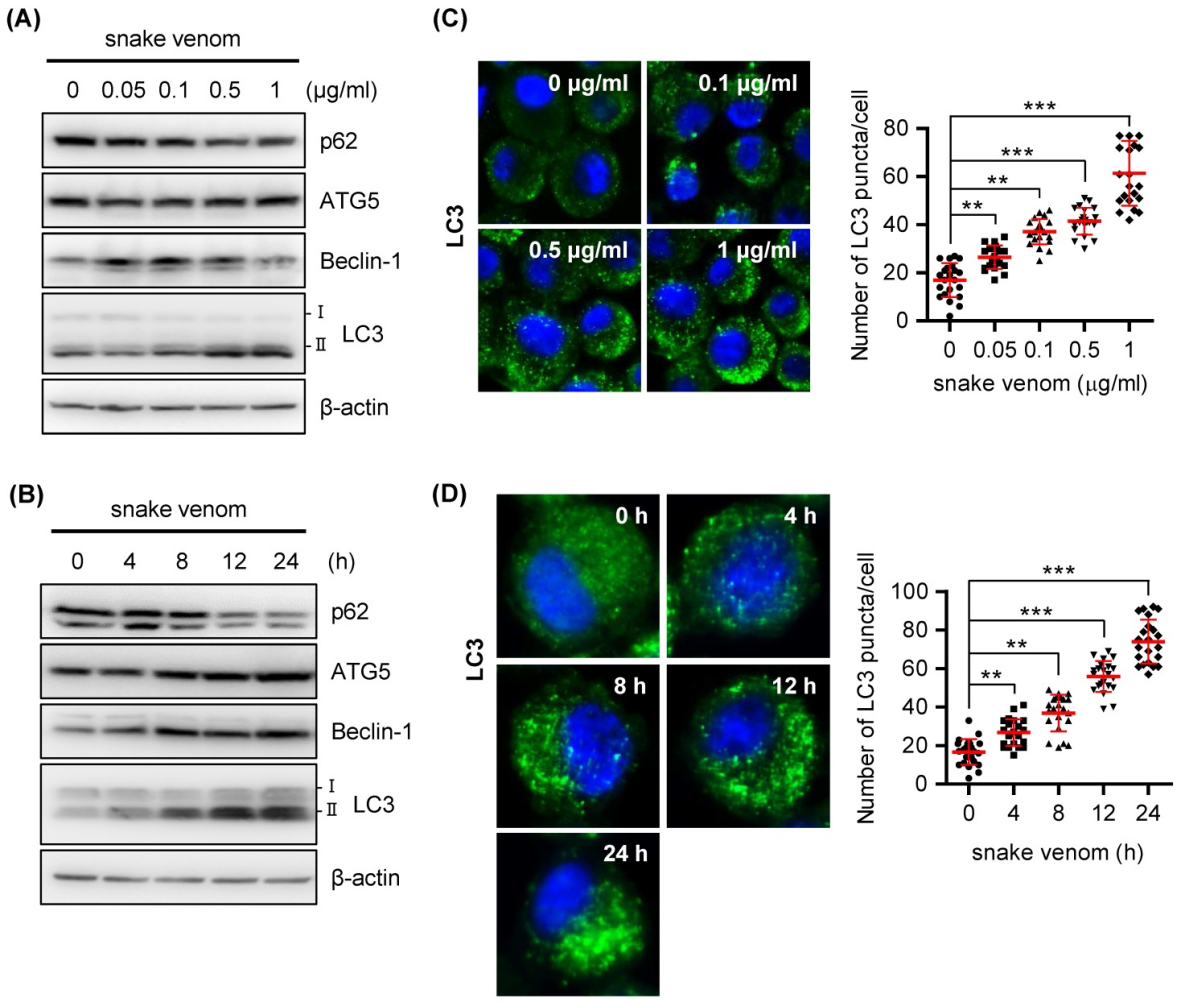
** Snake venom induces autophagy in a dose- and time-dependent manner.** (A) SW480 cells were treated with snake venom (0.05, 0.1, 1, 0.5, and 1 μg/ml) for 24 h. The indicated protein levels were evaluated by immunoblotting. (B) SW480 cells were treated with 1 μg/ml snake venom for the indicated time. The indicated protein levels were evaluated by immunoblotting. (C) SW480 cells were treated with snake venom (0.1, 0.5, and 1 μg/ml) for 24 h. Using fluorescent microscopy, LC3 puncta formation was detected after snake venom treatment. The percentage of cells with the LC3 puncta was calculated. (D) SW480 cells were treated with 1 μg/ml snake venom for the indicated time. Using fluorescent microscopy, LC3 puncta formation was detected after snake venom treatment. The percentage of cells with LC3 puncta was calculated.

Numerous studies have reported on the mode of cooperation between autophagy and apoptosis 17-19; both of these can be activated by multiple stressors, share multiple regulatory molecules, and also coordinate with each other. The results of our previous studies revealed that snake venom inhibits cell growth and induces apoptotic cell death in human CRCs 6. In this study, we attempted to investigate whether snake venom affects autophagy in CRCs. To investigate whether snake venom induces autophagy in SW480 cells, we examined the levels of the conversion of Microtubule-associated protein 1A/1B-light chain 3 (LC3)-II an autophagosome marker. Western blotting results showed that snake venom increased the protein levels of beclin-1, ATG5, and conversion of LC3-I to LC3-II, whereas the p62 level decreased in an obvious dose- and time-dependent manner after treatment with snake venom (Figure 1A and Figure 1B). To further confirm snake venom-induced autophagy, we stained the SW480 cells with LC3 antibodies and examined whether snake venom induces LC3 puncta formation, a marker of autophagosome formation. As shown in Figure 1C and Figure 1D, punctate fluorescence was observed in the snake venom-treated LC3-expressing SW480 cells; moreover, snake venom strongly induced autophagic cells in a dose- and time-dependent manner. We further whether snake venom induces autophagy in normal cell lines. Conversion of LC3-I to LC3-II was not changed in snake venom treated-CCD-18Co colon normal cells (Supplementary Figure S1A). In addition, LC3 puncta was not significantly different in snake venom treated CCD-18Co normal cell lines (Supplementary Figure S1B). To determine whether snake venom induces autophagy in other cancer cell lines, we confirmed the efficacy of snake venom against A549 lung cancer cells, HeLa CCL2 cervical cancer cells, and U-2 OS osteosarcoma cells; snake venom treatment increased the level of LC3-II and LC3 puncta in some of these cancer cell lines (Supplementary Figure S2A and S2B). Taken together, our data indicate that snake venom induced an autophagy in various cancer cell lines, particularly in SW480 CRCs.

### Snake venom induces autophagy flux

**Figure 2:**
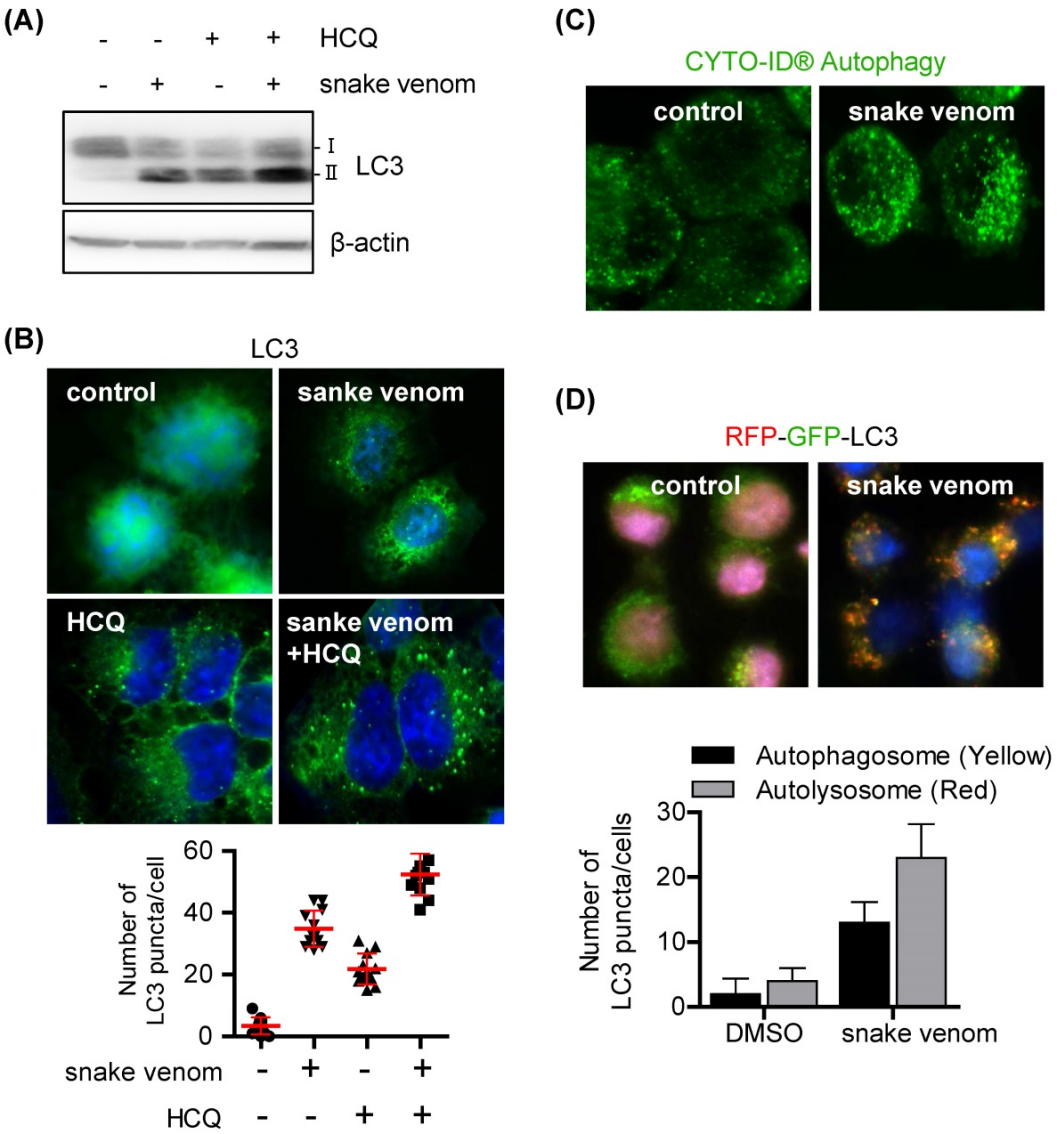
** Snake venom induces autophagic flux in lung cancer cells.** (A and B) SW480 cells were treated with 1 μg/ml snake venom for 24 h in the absence or presence of the lysosomal inhibitor HCQ (5 μM) for 3 h. LC3 levels were evaluated by immunoblotting (A) or immunocytochemistry (B). The nuclei were counterstained with DAPI. (C) SW480 cells were treated with 1 μg/ml snake venom and were then stained with CYTO-ID for 1 h at 37°C in the dark. The nuclei were counterstained with DAPI. (D) SW480 cells were incubated with RFP-GFP-LC3B for 24 h and then treated with 1 μg/ml snake venom. The nuclei were counterstained with DAPI.

Autophagy induction or autophagic flux blockade can increase LC3-II levels and autophagosome formation. To evaluate whether snake venom induces autophagic flux, cells were treated with snake venom with or without the autophagy inhibitor hydroxychloroquine (HCQ). HCQ increases LC3-II levels by inhibiting the fusion of autophagosomes and lysosomes to block autophagosome degradation. Therefore, increases in the LC3-II levels in the presence of HCQ are indicative of an enhanced autophagic flux. The LC3-II levels increased after treatment with snake venom or HCQ alone. Treatment with snake venom in the presence of HCQ further increased the LC3-II levels (Figure 2A). Furthermore, SW480 cells treated with snake venom, exhibited extensive distribution of fluorescent punctate patterns, which was also augmented by HCQ (Figure 2B). We used the CYTO-ID autophagy staining probe that selectively labels autophagic vacuoles and specifically partitions those into the lysosomal compartment 20; the results showed that snake venom induced significantly more autophagic flux. The untreated cells did not show any significant CYTO -ID autophagy staining, indicating that the probe only stained autolysosomes, thereby confirming active autophagic flux (Figure 2C). To further validate our findings, we used an LC3 tandem fluorescence construct that allows discrimination between autophagosomal and autolysosomal LC3. The SW480 cells were treated with snake venom. Untreated SW480 cells displayed bright, diffuse green and red fluorescence signals. The SW480 cells treated with snake venom displayed enhanced autophagosome formation LC3 (colocalization of green and red fluorescence) and a parallel increase in autolysosomal (only red fluorescence) (Figure 2D). These data suggest that the autophagosomal/autolysosmal pathway remains fully active in CRCs during treatment with snake venom.

### Snake venom-induced autophagic cell death

**Figure 3:**
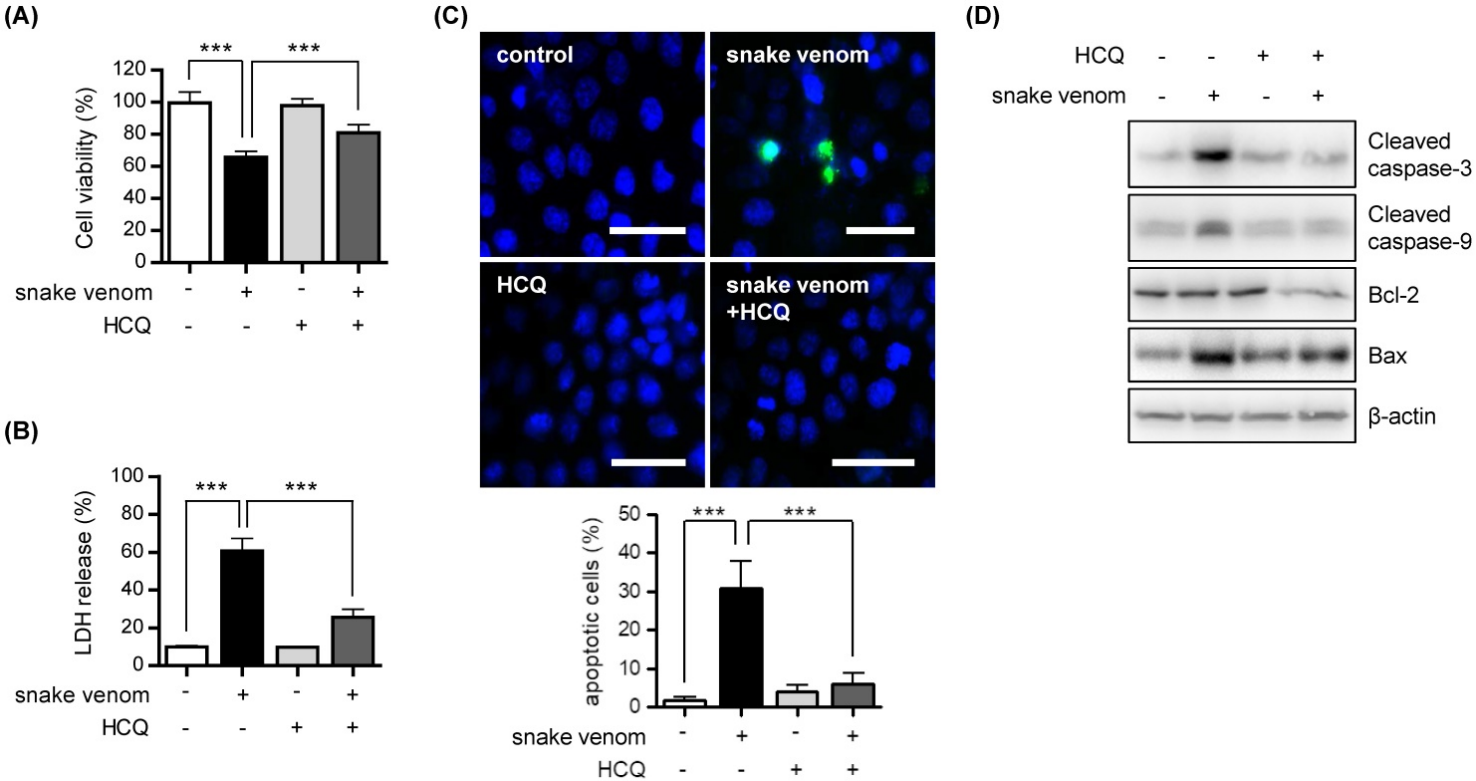
** Inhibition of autophagy reduced snake venom-induced cell death.** (A and B) SW480 cells were treated with 1 μg/ml snake venom for 24 h in the absence or presence of lysosomal inhibitor HC Q (5 μM). The viability of the snake venom- treated cells was measured by MTT assay (A) or LDH release assay (B). The data are presented as mean ± SD from three independent experiments. *** *P* < 0.05. (C) Representative fluorescence microscopy images showing nuclear staining for DAPI (blue) and TUNEL positive (Ggreen) in cells treated with 1 μg/ml snake venom for 24 h in the absence or presence of the autophagy inhibitor HCQ (5 μM). Scale bar, 50 μm. The number of stained positive cells was counted in three different fields and averaged. Data are presented as mean ± SD from three independent experiments. *** *P* < 0.05. (D) SW480 cells were treated with 1 μg/ml snake venom for 24 h in the absence or presence of HCQ (5 μM). The indicated protein levels were evaluated by immunoblotting.

In the context of cancer treatment, autophagy can promote either tumor cell survival or death 12, 14. To determine which effect is applicable to snake venom treatment, we exposed SW480 cells to HCQ. We found that cell viability significantly increased after snake venom treatment in the presence of HCQ as compared with snake venom treatment alone (Figure 3A). Moreover, compared with that with snake venom treatment alone, the release of lactate dehydrogenase (LDH) was reduced in with snake venom treatment in the presence of HCQ (Figure 3B). Our results showed that HCQ inhibited snake venom-induced cell apoptosis, as evidenced by the reduced number of TUNEL-positive apoptotic cells (Figure 3C). Furthermore, the results of western blotting showed that HCQ suppressed the snake venom treatment-mediated upregulation of the apoptosis-related protein levels (Figure 3D). Our data indicate that the suppression of autophagy blocks snake venom-induced apoptosis in SW480 cells.

### Snake venom induced autophagy via the JNK pathway

**Figure 4:**
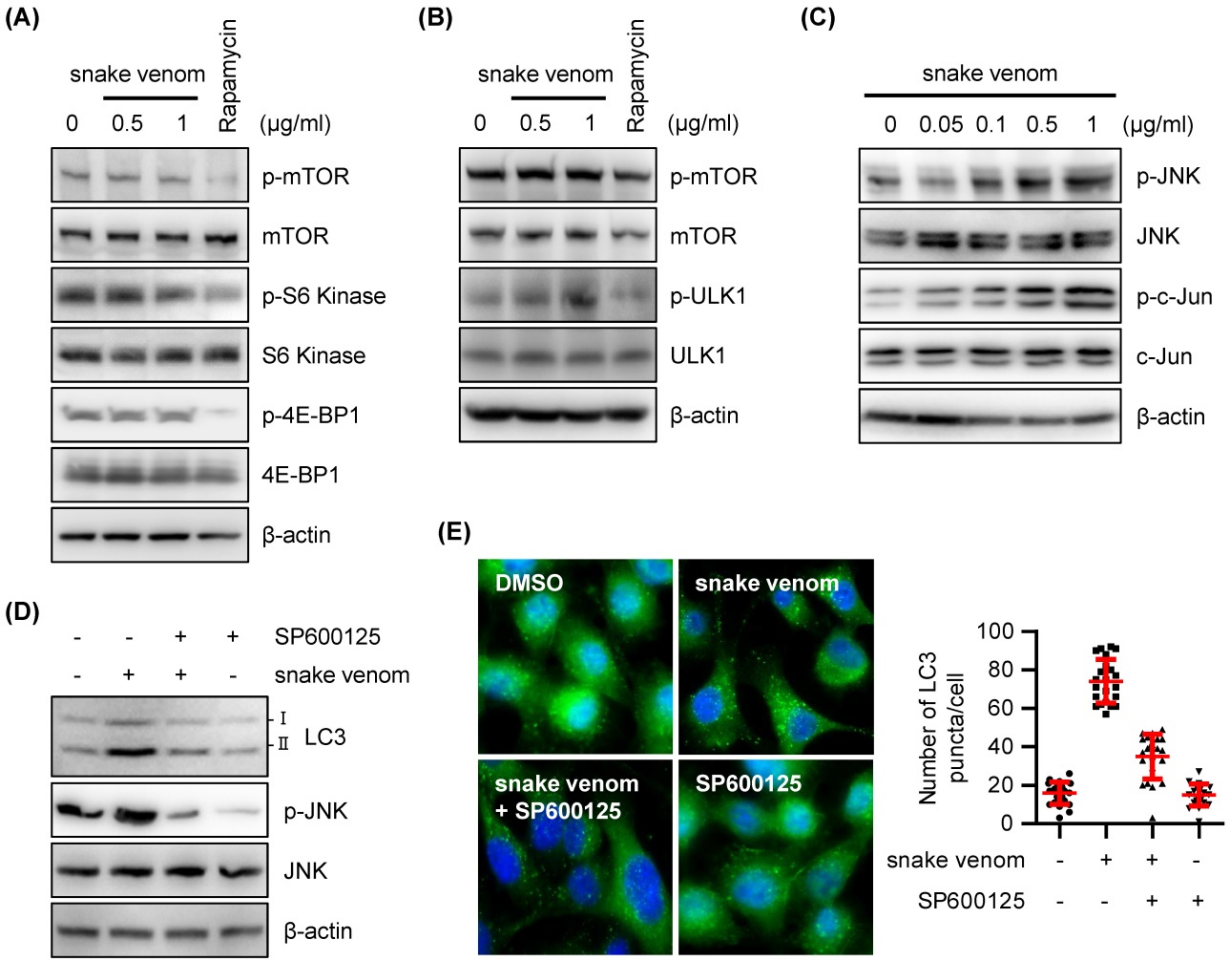
** The JNK signaling pathway participated in snake venom-induced autophagy.** (A and B) SW480 cells were treated with snake venom (0.5, 1 μg/ml) or 0.5 μM Rapamycin for 24 h. The indicated protein levels were evaluated by immunoblotting. (C) SW480 cells were treated with snake venom (0.5, 1 μg/ml) for 24 h. The indicated protein levels were evaluated by immunoblotting. (D) SW480 cells were treated with 1 μg/ml snake venom for 24 h in the absence or presence of JNK inhibitor SP600125 (20 μM) pretreated for 2 h. The LC3 levels were evaluated by immunoblotting (A) or immunocytochemistry (B). The nuclei were counterstained with DAPI.

To explore the mechanisms underlying snake venom-induced autophagy, we examined the effects of snake venom on phosphorylated mammalian target of rapamycin (mTOR) protein levels. mTOR is a serine/threonine kinase that belongs to the PK3K-related family kinase that has been known to regulate cell growth, cell proliferation, autophagy, and protein synthesis through growth factors, nutrients, and stress stimulation 21-24. Unexpectedly, the phosphorylation levels of mTOR remained unchanged, suggesting that mTOR is not required for snake venom-induced autophagic response (Figure 4A). Snake venom did not affect the phosphorylation of the mTOR downstream targets p70 ribosomal protein S6 kinase (p70S6K Thr389, Ser371), eukaryotic translation initiation factor 4E binding protein 1 (4E-BP1, Thr 37/46), and ULK (Figure 4A and 4B), indicating that snake venom induces autophagy via an mTOR-independent pathway.

JNK is reportedly involved in mTOR-independent autophagy 25-28. Therefore, JNK activation following snake venom treatment has been investigated. We found that the levels of activated JNK are increased upon snake venom treatment in a dose-dependent manner (Figure 4C). Indeed, JNK activation correlated with an increase in c-Jun phosphorylation levels (Figure 4C). Blocking JNK using the pharmacological inhibitor SP600125 attenuated the expression levels of LC3-II after snake venom treatment (Figure 4D). LC3 puncta formation was considerably decreased in the cells treated with a JNK inhibitor combined with snake venom as compared with snake venom alone (Figure 4E). The JNK inhibitor alone did not cause greater puncta formation in treated versus untreated cells. These data indicate that JNK signaling is a critical regulator of snake venom-induced autophagy.

### Snake venom induced autophagic cell death

**Figure 5:**
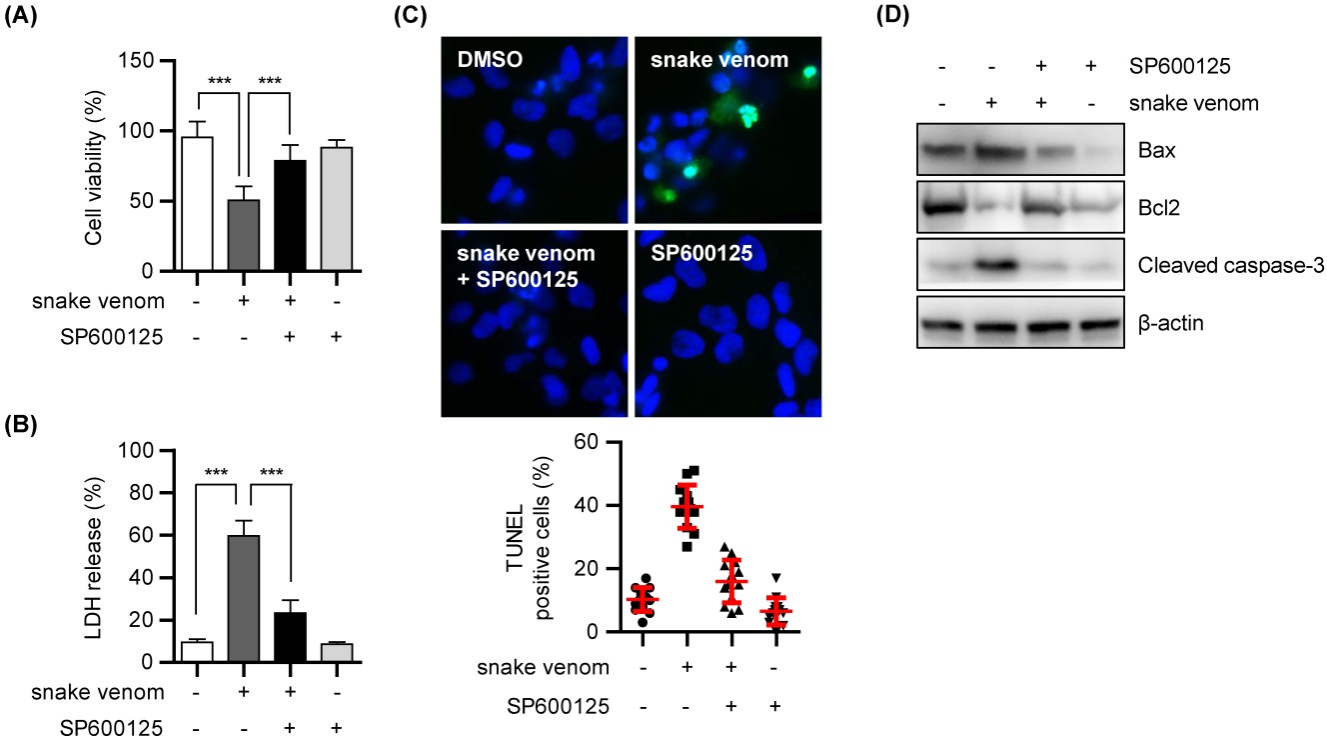
** Inhibition of JNK activity reduced snake venom-induced cell death.** (A and B) SW480 cells were treated with 1 μg/ml snake venom for 24 h in the absence or presence of JNK inhibitor SP600125 (20 μM) pretreated for 2 h. The cell viability of snake venom treated cells was measured by MTT assay (A) or LDH release assay (B). The data are presented as mean ± SD in three independent experiments. *** *P* < 0.05. (C) Representative fluorescence microscopy images showing nuclear staining for DAPI (blue) and TUNEL positive (Green) in cells treated with 1 μg/ml snake venom for 24 in the absence or presence of JNK inhibitor SP600125 (20 μM) pretreated for 2 h. The number of stained positive cells were counted in three different fields and averaged. The data are presented as mean ± SD in three independent experiments. *** *P* < 0.05. (D) SW480 cells were treated with 1 μg/ml snake venom for 24 h in the absence or presence of JNK inhibitor SP600125 (20 μM) pretreated for 2 h. The indicated protein levels were evaluated by immunoblotting.

Next, we investigated whether snake venom-induced autophagic cell death depends on JNK pathway activation. To address this, SW480 cells were treated with SP600125 with or without snake venom. SP600125 hindered the cytotoxic effects of snake venom (Figure 5A and 5B). Moreover, the TUNEL assay further indicated that treatment with SP600125 prevents the snake venom-induced increase in the apoptotic rate in SW480 cells (Figure 5C). In addition, the results of western blotting demonstrated that both the treatments suppressed the levels of apoptosis-related proteins (Figure 5D). Taken together, our results suggested that snake venom induces colorectal cell apoptosis and autophagy via the JNK signaling pathway.

## Discussion

Our data demonstrate that snake venom stimulates and induces autophagy, as indicated by the increased conversion of LC3-I to lapidated LC3-II, the degradation of p62, and the microscopic evaluation of LC3 puncta formation. Furthermore, we found that snake venom induced autophagy via JNK activation and by triggering apoptosis through the JNK signaling pathway. Our data demonstrate that snake venom-induced autophagy promotes apoptosis in CRCs and might become a new approach for the treatment of colorectal cancer.

The LC3 system plays an important role in autophagosome maturation; LC3-II attaches to the isolation membrane during the elongation stage and, unlike the Atg12-Atg5-Atg16 complex, remains attached until it degrades 29. The protein p62 has an ubiquitin-associated domain that can bind to ubiqutinated proteins. The ubiqutinated protein-p62 complex interacts with the LC3-II attached to the autophagosome and, eventually, the complexes and organelles in the autophagosome containing p62 get degraded by lysosomes 12. In the present study, we first demonstrated that snake venom induced autophagy in SW480 cells. Snake venom-induced autophagy was reported on the basis of LC3 conversion and accumulation of the LC3 punctate formation pattern. Moreover, snake venom decreased the p62 protein level in a dose- and time-dependent manner as demonstrated using western blotting. But snake venom was not induced autophagy in CCD-18Co normal colon cells. We showed that snake venom induced autophagy in A549 lung cancer cells, HeLa CCL2 cervical cancer cells, and U-2 OS osteosarcoma cells. These findings indicate that snake venom could generally induce autophagy in various cancer cell lines not normal cell lines. We used pharmacological approaches to inhibit autophagy. The results of the western blotting of LC3-II protein expression under treatment with HCQ showed that autophagy can increase after exposure to snake venom.

Autophagy and apoptosis are not completely independent of each other, they are associated with each other through intracellular signaling systems. However, unlike apoptosis, autophagy not only leads to cell death but also exerts ambivalent functions that contribute to cell survival 12, 14. In our previous study, we demonstrated that snake venom induces apoptosis; in this study, we found that snake venom also induces autophagy. The expression of autophagy- and apoptosis-related proteins was indeed reduced after combined treatment with snake venom and HCQ in CRCs. Our data thus suggest that snake venom induces autophagic cell death.

Autophagy is induced by various signaling pathways and is promoted by the down-regulation of mTOR, a representative central metabolic sensor 30-32. However, our results showing that the levels of mTOR substrates remained unchanged in snake venom-treated cells suggest that snake venom-induced autophagy is mTOR-independent. JNK signaling is critical for the induction of mTOR-independent autophagy. JNK regulates cell proliferation, cell differentiation, apoptosis, and autophagy and is activated by extracellular signals 25-27, 33. Hence, the phosphorylation of JNK was increased in CRCs treated with snake venom. Inhibition of JNK by SP600125 reduced the LC3-II levels in snake venom treated-SW480 cells. These results demonstrated that the activation of the JNK pathway by snake venom is critical to snake venom-induced autophagy. Moreover, the activation of JNK by snake venom affects autophagy-induced apoptosis. Inhibition of the JNK activity by SP600125 also decreased the levels of cleaved caspase-3 and -9, which may lead to snake venom induced autophagy via the JNK signaling pathway.

## Conclusion

We demonstrated that snake venom can induce autophagy in various cancer cell lines. In addition, the activation of the JNK signaling pathway is important for snake venom-induced autophagy. Importantly, SP600125 inhibited autophagy yet suppressed snake venom-induced apoptosis, indicating that it is a key requirement of the JNK pathway for increasing of autophagic cell death via snake venom treatment. These data indicate that snake venom-mediated autophagy is important for apoptosis and that the JNK signaling pathway plays a role in the underlying mechanism. Our study findings suggest that snake venom has apoptotic effects and autophagy-induced activity in cancer cells and can be further developed as an anticarcinogen.

## Supplementary Material

Supplementary figures.

## Abbreviations

Colorectal cancer cells: CRCs
Microtubule-associated protein 1A/1B-light chain 3: LC3
Hydroxychloroquine: HCQ
Mammalian target of rapamycin: mTOR
p70 ribosomal protein S6 kinase: p70S6K
4E binding protein 1: 4E-BP1
