# Supplementary material for: Snake venom induces an autophagic cell death via activation of the JNK pathway in colorectal cancer cells
Source: J Cancer. 2022 Sep 21;13(12):3333–41. doi: 10.7150/jca.75791 (PMC9516008; doi:10.7150/jca.75791)
Supplement: Supplementary file 1 — Supplementary figures. [file jcav13p3333s1.pdf]

Supplementary materials

Supplementary Figure S1

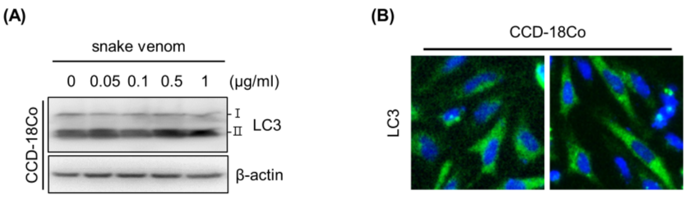

Supplementary Figure S1. Snake venom not induces autophagy in normal colon cells.

(**A**) CCD-18Co cells were treated with snake venom (0.05, 0.1, 0.5, and 1 μg/ml) for 24 h. LC3 levels were evaluated by immunoblotting. (**B**) Cells were treated with 1 μg/ml snake venom for 24 h. The formation of LC3 puncta was detected after snake venom treatment under a fluorescence microscope.

Supplementary Figure S2

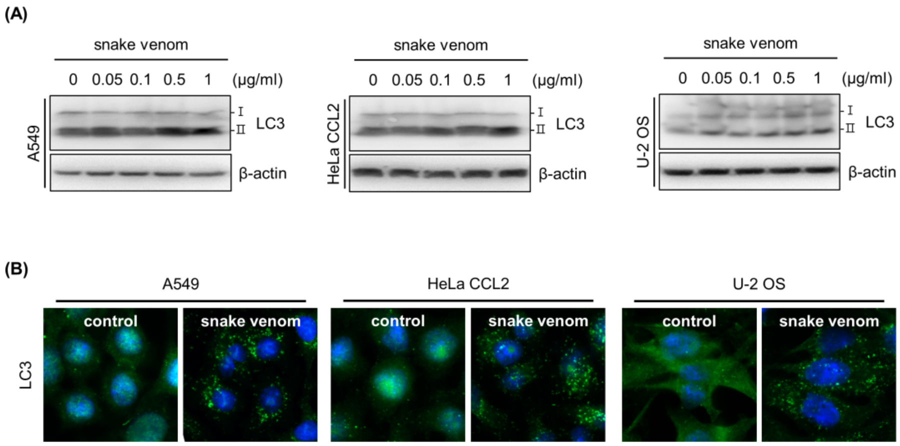

Supplementary Figure S2. Snake venom induces autophagy in various cancer cell lines.

(**A**) Cells were treated with snake venom (0.05, 0.1, 0.5, and 1 μg/ml) for 24 h. LC3 levels were evaluated by immunoblotting. (**B**) Cells were treated with 1 μg/ml snake venom for 24 h. The formation of LC3 puncta was detected after snake venom treatment under a fluorescence microscope.
